# Fisetin regulates the biological effects of rat nucleus pulposus mesenchymal stem cells under oxidative stress by sirtuin‐1 pathway

**DOI:** 10.1002/iid3.865

**Published:** 2023-05-16

**Authors:** Qing Zhou, Chao Zhu, Anwu Xuan, Junyou Zhang, Zhenbiao Zhu, Liang Tang, Dike Ruan

**Affiliations:** ^1^ Navy Clinical College Anhui Medical University Hefei Anhui China; ^2^ The Fifth School of Clinical Medicine Anhui Medical University Hefei Anhui China; ^3^ Department of Orthopedic Surgery The Sixth Medical Center of PLA General Hospital Beijing China; ^4^ The Second School of Clinical Medicine Southern Medical University Guangzhou China

**Keywords:** cells, apoptosis, inflammation, processes, stem cells

## Abstract

**Background:**

Excessive oxidative stress has been accepted as one of the critical factors for intervertebral disc degeneration (IDD), which is associated with low back pain (LBP). Fisetin (Fis) is a bioactive flavonoid that possesses strong bioactive activity. In present study, we aimed to illuminate the role of Fis on nucleus pulposus mesenchymal stem cells (NPMSCs).

**Methods:**

NPMSCs were isolated and cultured from rat NP tissues and identified by flow cytometry and multilinear differentiation. The cytotoxicity of Fis, EX‐527, and hydrogen peroxide (H_2_O_2_) on NPMSCs was validated using Cell Counting Kit‐8 tests. Cell apoptosis was tested by flow cytometry and TUNEL assay. Inflammatory mediators were assessed by Elisa tests, RT‐PCR. Extracellular matrix (ECM) metabolism was measured by Western blot analysis and RT‐qPCR. The expression of the SIRT1 was evaluated by Western blot analysis.

**Results:**

NPMSCs were successfully isolated and cultured from rat NP tissues, and it has been identified by flow cytometry and multilinear differentiation. The results showed that Fis attenuated H_2_O_2_‐induced apoptosis, inflammation, and ECM degradation of NPMSCs. Moreover, the above protective effects of Fis can be inhibited by EX‐527, a unique SIRT1 inhibitor, indicating that SIRT1 may involve in the mechanism of Fis in protecting NPMSCs from oxidative stress.

**Conclusions:**

As a natural compound with little cytotoxicity on NPMSCs, Fis alleviate H_2_O_2_‐induced apoptosis, inflammation, and ECM degradation by suppressing oxidative stress, this finding may add the theoretical basis for research on new treatment of IDD based on NPMSCs.

## Graphical abstract

Fisetin (Fis) could attenuate cell viability decrease, inflammation, cell apoptosis, and extracellular matrix degradation induced by H_2_O_2_ in nucleus pulposus mesenchymal stem cells (NPMSCs) by activating SIRT1. Inhibiting SIRT1 reversed the protection of Fis on NPMSCs exposed to H_2_O_2_.

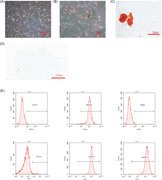

## 1 INTRODUCTION

Low back pain (LBP) is a frequent musculoskeletal disorder that severely affects patients' quality of life and causes an enormous economic burden on society.^1^ It has been reported that approximately 80% of people worldwide suffer from LBP at least once during their lifetime.^2^ Intervertebral disc degeneration (IDD) is thought to be the major cause of LBP.^3^ However, no effective therapies truly reverse or inhibit the process of IDD.

Oxidative stress is generally considered a key factor during the process of IDD.^4^ Previous studies have strongly suggested that abnormal and excessive reactive oxygen species (ROS) production may lead to oxidative stress damage and even cell death.^5^ Additionally, it has been confirmed that the excessive cell apoptosis caused by oxidative stress contributes to IDD.^6^ Regarding the excessive oxidative stress in IDD development, it has attracted the attention of researchers in IDD.

Stem cell therapy has been regarded as promising management of IDD and is getting increased interest for its potential clinical applicability.^7^ However, achieving the survival rate of translated stem cells in inner IVD is one of the biggest challenges. Nucleus pulposus mesenchymal stem cells (NPMSCs), one type of mesenchymal stem cell found in IVD, have a higher ability to survive in an acidic microenvironment.^8^ In the present context, NPMSCs may be considered an appealing and ideal cell target in the IDD study.

3,7,3,4‐tetrahydroxyflavone (fisetin, Fis), a bioactive flavonoid that has been found in many fruits and vegetables, including apples, grapes, and onions.^9^, ^10^ Many studies demonstrated that Fis has antioxidant, anti‐inflammatory, and antitumor biological activities.^11^, ^12^ However, it has not been illuminated that the protective activities of Fis on oxidative stress in NPMSCs. Thus, we examined the antioxidant effect of Fis on NPMSCs and the potential pathway of Fis in NPMSCs under H_2_O_2_‐stimulation.

## 2 MATERIALS AND METHODS

### 2.1 Primary rat NPMSCs

An overdose of pentobarbital was used to euthanize 20 random Sprague–Dawley rats. Type II collagenase, 2 mg/mL−0.1%, was used to digest the collected tissues at 37°C for 4 h. The digested tissues were then transferred to (DMEM)/F12 (Gibco) with 10% fetal bovine serum (FBS; Gibco) and antibiotics (1% penicillin/streptomycin) in an incubator at 37°C maintaining 5% CO_2_. A first medium change was performed after 48 h of incubation. NPMSCs were harvested when cells confluence to 80%–90%. The first two passages were used for experiments. All animal experiments were approved by Institutional Animal Care and Use Committee (approval number AWE2021122401). The experimental process strictly followed the approved protocol.

### 2.2 Identification of NPMSCs

The surface marker expression of NPMSCs isolated from rats was analyzed using flow cytometry, including CD11b, CD44, CD34, CD45, CD90, and CD29. Briefly, NPMSCs were stained with rabbit anti‐rat polyclonal antibodies and analyzed by flow cytometry (BD FACSCanto™).

NPMSCs were cultured into a special inducing medium (Cyagen) for 3 weeks to evaluate the osteogenic, adipogenic, and chondrogenic differentiation potential. Finally, alizarin red (Cyagen), oil red O (Cyagen), and alcian blue staining (Cyagen) were performed, respectively, according to the instructions of the manufacturer.

### 2.3 Cell viability analysis

To detect the viability of NPMSCs, Cell Counting Kit‐8 (CCK‐8) assaying was performed (CCK‐8; Dojindo) according to the manufacturer's instructions. First, the NPMSCs were seeded in 96‐well plates at a density of 5000 cells/well for 24 h. NPMSCs were separated and treated with normal basal medium, Fis or EX‐527, for 24 h and treated with H_2_O_2_ at different concentrations for 8 h. Then, 100 μL DMEM/F12 and 10 μL CCK‐8 solutions were supplemented to each well. The cells were then cultured for 2 h at 37°C. Finally, the OD value was determined at 450 nm using a microplate reader (BioTek).

### 2.4 TNF‐α and IL‐6 measurement

NPMSCs were cultured in six‐well plates at a density of 2 × 10^4^ cells/well and incubated with Fis for 24 h before H_2_O_2_ stimulation for 8 h. According to the manufacturer protocol, we measured TNF‐α and IL‐6 levels in the supernatants of each sample using an ELISA kit (Beyotime).

### 2.5 Real‐time PCR

NPMSCs were cultured in a six‐well plate. RNA was extracted from NPMSCs using Trizol reagent (Invitrogen) according to the manufacturer's instructions. A reverse transcriptase (TaKaRa) was used to synthesize complementary DNA (cDNA) from total RNA. Then, an SYBR Premix Ex Tag kit (TaKaRa) was used for RT‐PCR. During cycling, 40 cycles of denaturation at 95°C for 5 s were followed by 24 s of amplification at 60°C. As a loading control, glyceraldehyde 3‐phosphate dehydrogenase (GAPDH) was used. Table 1 lists PCR primers.

**Table 1:** Primer sequence for gene used in this present study.

Gene	F	R
IL‐6	CCGGGAGCAAAGGTCTA	CCGGGGAACTCTGACAT
TNF‐α	GTCAGATCATCTTCTCGAACC	CAGATAGATGGGCTCATACC
Aggrecan	TCCGCTGGTCTGATGGACAC	CCAGATCATCACTACGCAGTCCTC
Collagen II	TCCTAAGGGTGCCAATGGTGA	AGGACCAACTTTGCCTTGAGGAC
MMP3	TTTGGCCGTCTCTTCCATCC	GCATCGATCTTCTGGACGGT
MMP13	TGAAGTAGGACTGGGCAGAGA	TTTGGGTCAGGTGTCCACTC

### 2.6 Western blot

The protein expression of Collagen II (COL2), aggrecan (ACAN), matrix metalloproteinase 3 (MMP3), and matrix metalloproteinase 13 (MMP13) in NPMSCs was measured using Western blot analysis. Proteins were extracted from the NPMSCs with RIPA, and the concentration was tested as described in the protocols of the BCA Protein Assay Kit. SDS‐PAGE was used to separate protein at 120 V for 30 min and 90 V for 90 min. After that, proteins were transferred onto polyvinylidene fluoride (PVDF) membranes at 400 mA for 2 h. The membranes were blocked for 5 min at room temperature. The following primary antibodies were incubated with the PVDF membranes at 4°C overnight: COL2, ACAN, MMP3, MMP13, and GAPDH. Finally, the PVDF membranes were treated with secondary antibodies for 2 h at room temperature, and the amount of protein expression was assessed using ECL.

### 2.7 TUNEL method

TUNEL assay was also performed using the colorimetric TUNEL apoptosis assay kit to confirm cell apoptosis according to the manufacturer's instructions (Beyotime) and consequently imaged using a biological microscope.

### 2.8 Flow cytometry analysis

classic Annexin V‐FITC/PI staining kit was used to determine the apoptotic rate of NPMSCs. First, the NPMSCs were collected from plates, washed with phosphate‐buffered saline for three times, and suspended in 100 μL binding buffer. Cell apoptosis was determined using a flow cytometer (BD Co.). Cell staining positive for Annexin V and negative for PI were considered apoptotic NPMSCs.

### 2.9 Statistical analysis

The data were presented as mean ± standard deviation (SD) and analyzed by SPSS 22 software. One‐way analysis of variance was used to compare multiple groups. Differences were considered statistically significant when *p* value was less than 0.05.

## 3 RESULTS

### 3.1 Isolation and identification of NPMSCs

**Figure 1:**
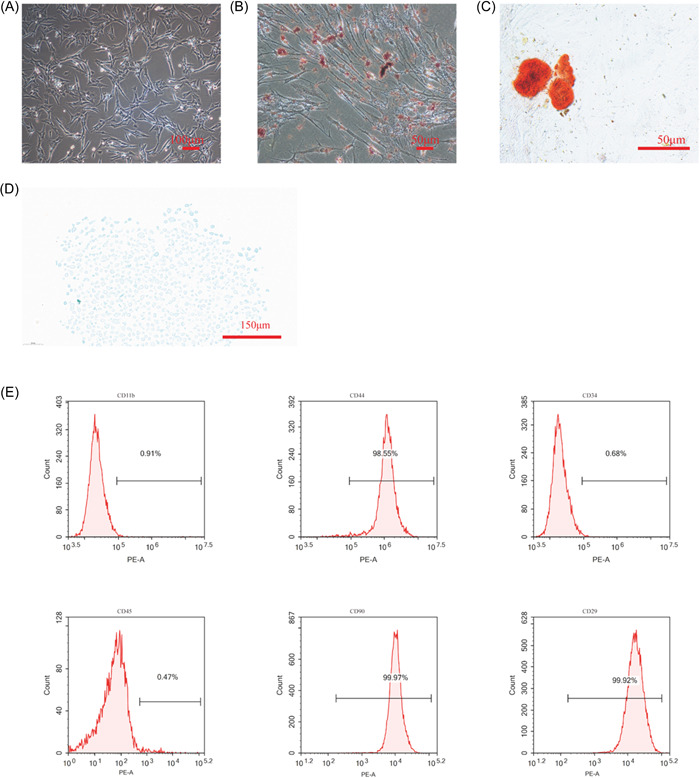
Isolation and culture of NPMSCs. (A) Third‐generation rat NPMSCs. (B) Oil red O staining indicated adipogenic differentiation. (C) Alizarin red staining indicated osteogenic differentiation. (D) Alcian blue staining indicated chondrogenic differentiation. (E) Flow cytometry analysis showed low expression of CD34, CD11b, and CD45, and high expression of CD44, CD90, and CD29. NPMSC, nucleus pulposus mesenchymal stem cell.

NPMSCs were isolated and cultured from rat NP tissue, observed the P2 generation NPMSCs using the microscope (Figure 1A). The surface markers on the NPMSCs were detected by Flow cytometry. We detected the multilineage differentiation of NPMSCs. After osteogenic induction, calcium junction deposits were observed in NPMSCs using Alizarin Red staining (Figure 1B). NPMSCs formed visible lipid droplets after adipogenic differentiation and were detected using Oil Red O staining (Figure 1C). Also, there are accumulated sulfated proteoglycans in NPMSCs after chondrogenic differentiation (Figure 1D). Furthermore, The expression rates were above 95%, which means that the surface markers CD were highly expressed, and our results indicated that negative markers CD did not exceed 5% (Figure 1E). The results above indicated rat NPMSCs had been successfully isolated and cultured.

### 3.2 Effects of Fis on NPMSCs viability

**Figure 2:**
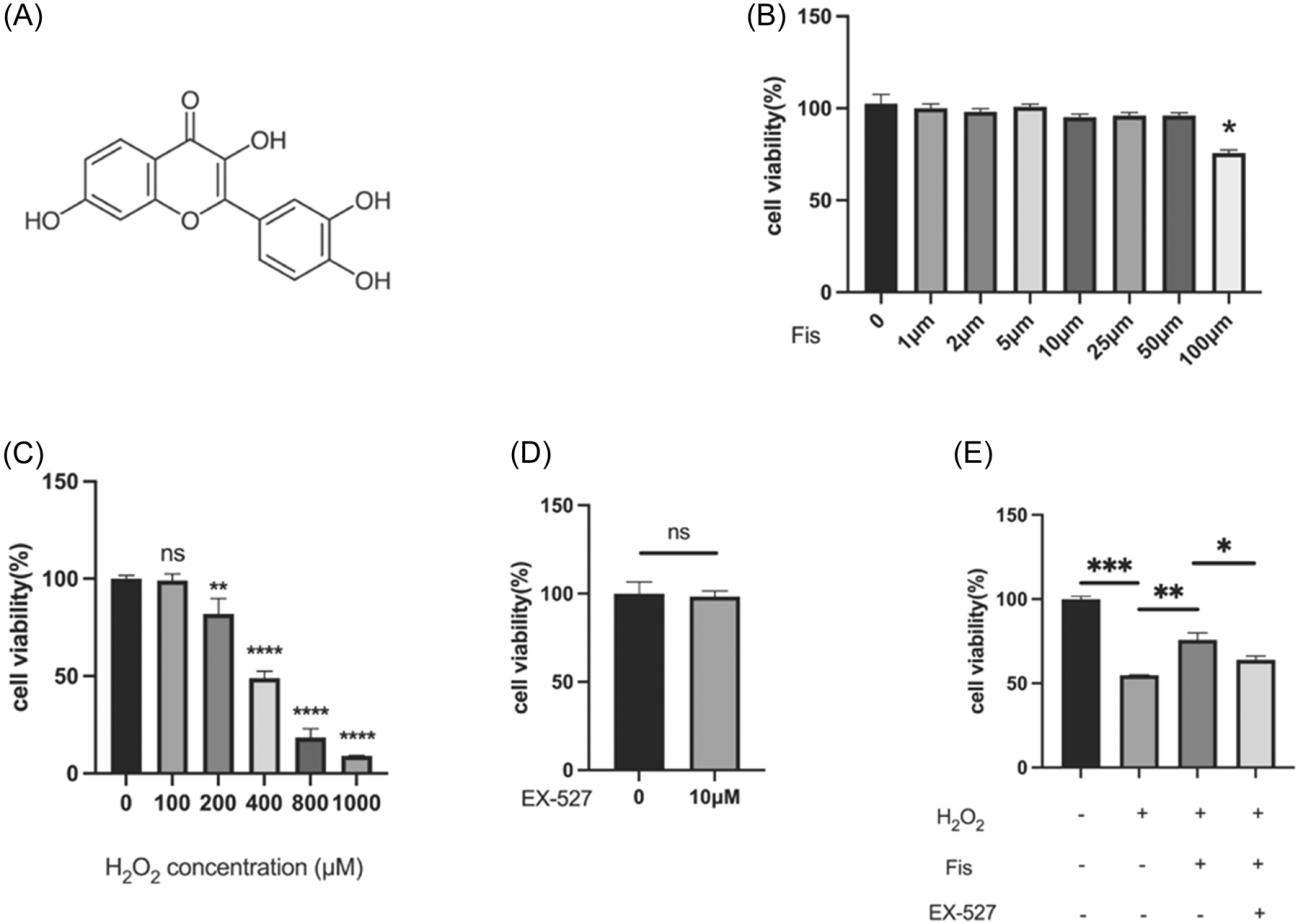
Elevated the effect of Fis on NPMSCs viability. (A) The chemical structure of Fis. (B) There was no obvious cytotoxic effect of Fis on NPMSCs when cells were exposed to 10 μM Fis. (C) The different concentration of H_2_O_2_ on NPMSCs viability (D) 10 μM EX‐527 was safe for NPMSCs. (E) The cell viability measured by CCK‐8. *p < .05, **p < .01, ***p < .001, ****p < .0001. CCK‐8, Cell Counting Kit‐8; Fis, Fisetin; NPMSC, nucleus pulposus mesenchymal stem cell; ns, no significance.

The chemical structure of Fis is shown in Figure 2A. Different concentrations of Fis were used to assess the cytotoxic effect of Fis on NPMSCs by CCK‐8 assay after incubating for 24 and 48 h. The viability of NPMSCs was unaffected by Fis under the concentration of 50 μM (Figure 2B). Therefore, we use the dose of 10 μM in the following experiments. Next, the effect of Fis on H_2_O_2_‐treated NPMSCs was evaluated. The NPMSCs were pretreated with Fis for 24 h and then stimulated with H_2_O_2_ for 8 h. As shown in Figure 2C, the results showed that 10 μM EX‐527 has no cytotoxicity on NPMSCs (Figure 2D). Treatment with H_2_O_2_ significantly reduced the cell viability of NPMSCs, and this effect can be attenuated by Fis pretreatment (Figure 2E).

### 3.3 Fis inhibit H_2_O_2_‐induced apoptosis in NPMSCs

**Figure 3:**
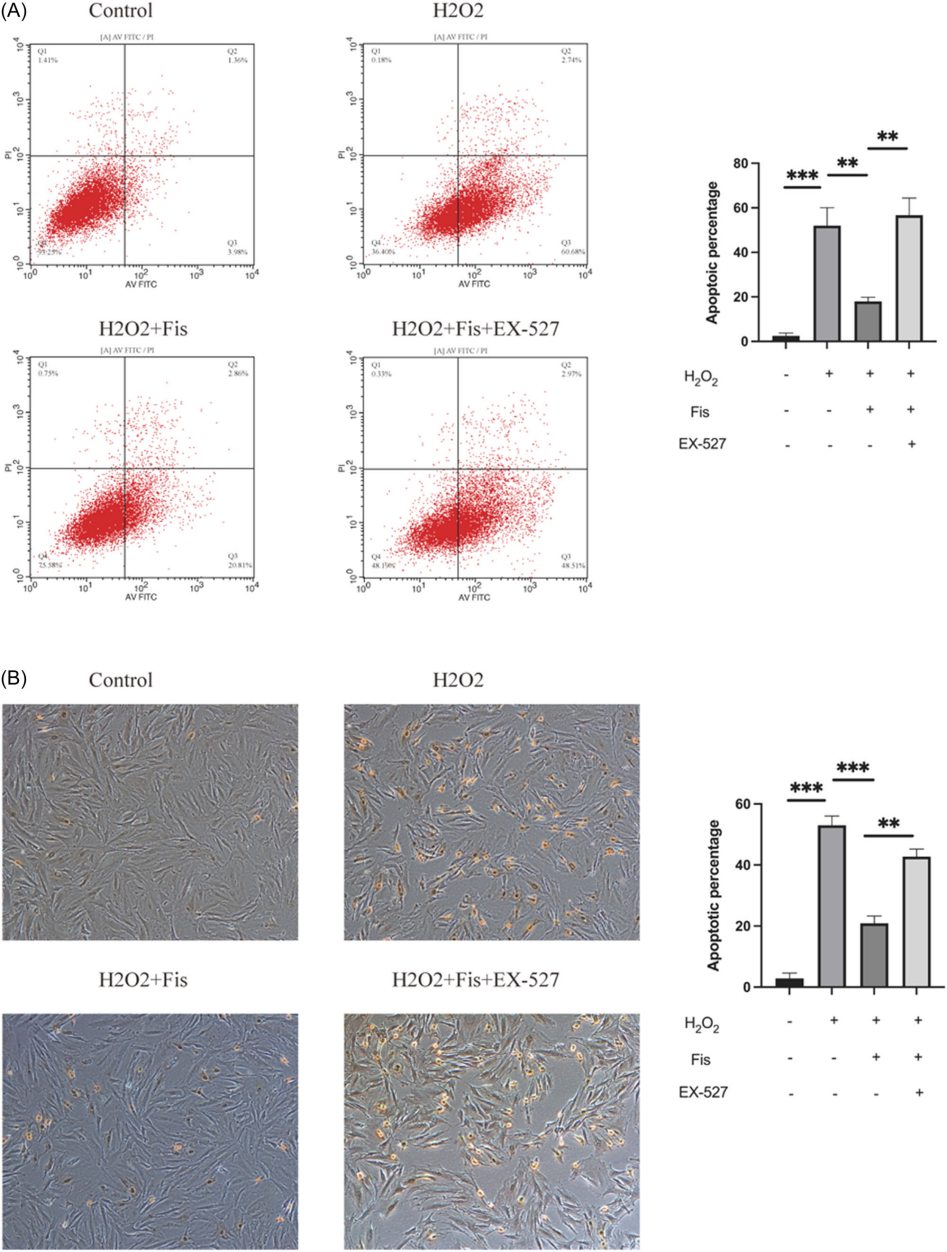
Elevated the protective role of Fis on NPMSCs apoptosis under oxidative stress. (A) The flow cytometry analysis showed that Fis alleviated H_2_O_2_‐induced NPMSCs apoptosis and was partly inhibited by EX‐527. (B) The TUNEL analysis showed that Fis alleviated H_2_O_2_‐induced NPMSCs apoptosis and was partly inhibited by EX‐527. *p < .05, **p < .01, ***p < .001, ****p < .0001. Fis, Fisetin; NPMSC, nucleus pulposus mesenchymal stem cell; ns, no significance.

NPMSCs were treated with 200 μM H_2_O_2_ for 8 h, the result tested by Annexin V‐FITC/PI staining showed that the apoptotic percentage of H_2_O_2_‐treated group was higher than the control group, and this effect can be partly attenuated by Fis (Figure 3A). Meanwhile, the TUNEL assay showed similar results (Figure 3B). These data demonstrated that Fis could significantly inhibit the apoptosis induced by NPMSCs.

### 3.4 Fis inhibit H_2_O_2_‐induced inflammation in NPMSCs

**Figure 4:**
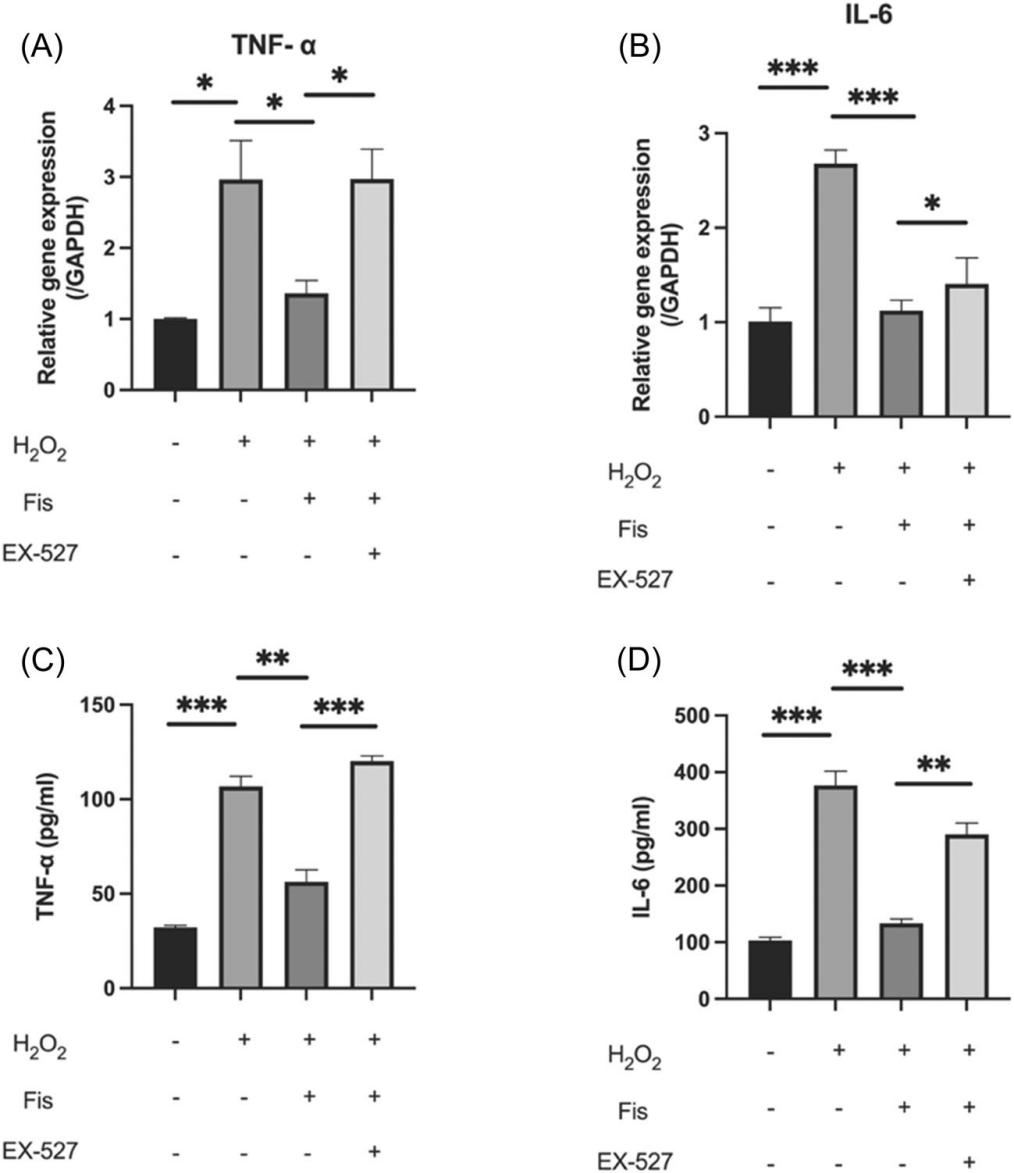
The results of the inflammation‐related level. (A–C) The gene expression of COX‐2, IL‐6, and TNF‐α showed that Fis can attenuate the inflammation induced by H_2_O_2_ in NPMSCs, and this protective effect can be partly inhibited by EX‐527. (D, E) The results of TNF‐α and IL‐6 level in culture medium supernatant. *p < .05, **p < .01, ***p < .001, ****p < .0001. NPMSC, nucleus pulposus mesenchymal stem cell; ns, no significance.

We explored the in‐depth potential mechanism of Fis inhibiting the H_2_O_2_‐induced inflammation in NPMSCs by testing the inflammation level using the Elisa experiment. The results showed that the IL‐6 and TNF‐α levels were highly increased, and this type of inflammation agent can be decreased by Fis stimulation (Figure 4A,B). Subsequently, we used RT‐PCR experiments to assay the inflammation‐related gene expression. And the results showed that the IL‐6 and TNF‐α were highly increased after being stimulated with H_2_O_2_. However, this effect could be partly inhibited by cocultured with Fis (Figure 4C,D). Overall, Fis relieved the inflammation induced by H_2_O_2_ in NPMSCs.

### 3.5 Fis inhibit H_2_O_2_‐induced extracellular matrix remodeling in NPMSCs

**Figure 5:**
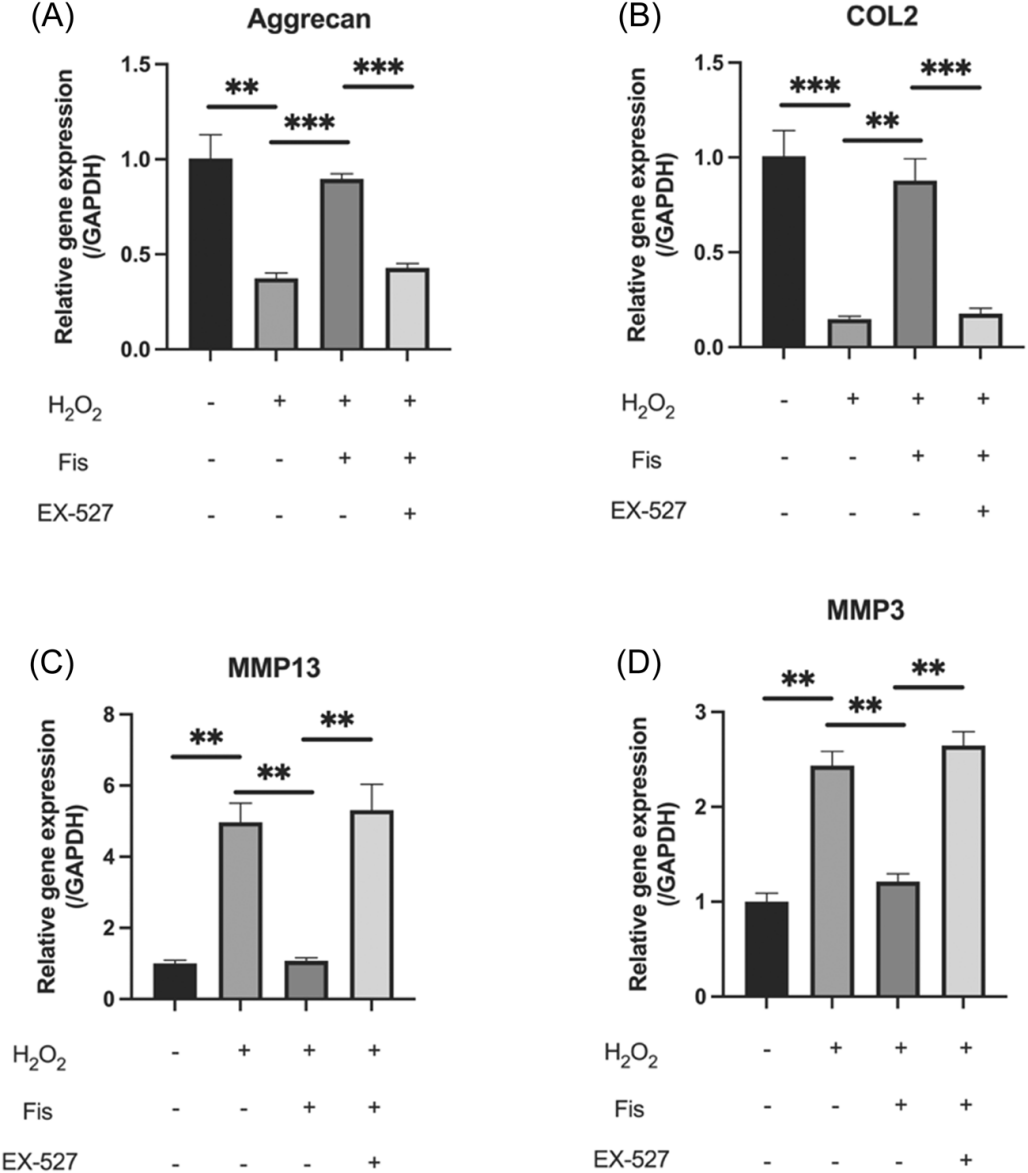
(A–D) The results of gene expression of aggrecan, COL‐2, MMP13, and MMP3 which reflected the level of ECM, it was showed that the gene expression of ECM related is reduced after H_2_O_2_ stimulated and increased after cultured with Fis. Finally, this protective effect can be partly inhibited by EX‐427. *p < .05, **p < .01, ***p < .001, ****p < .0001. ECM, extracellular matrix; ns, no significance.

**Figure 6:**
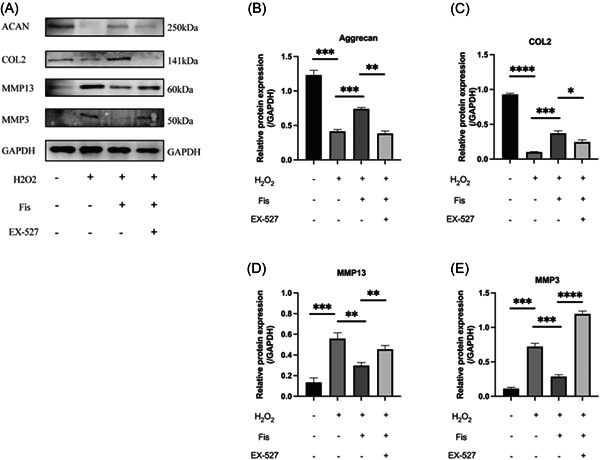
(A) The result of Western blot analysis. (B–E) The results of ECM‐related protein expression in NPMSCs, it showed that ECM‐related protein expression reduced after H_2_O_2_ was stimulated and increased after cultured with Fis. Finally, this protective effect can be partly inhibited by EX‐427. *p < .05, **p < .01, ***p < .001, ****p < .0001. NPMSC, nucleus pulposus mesenchymal stem cell; ns, no significance.

First, we determined the mRNA levels of aggrecan, COL2, MMP13, and MMP3 using an RT‐PCR experiment, and the results revealed that the related mRNA expression of extracellular matrix (ECM) was highly decreased after being stimulated with H_2_O_2_. However, this effect can be attenuated by Fis (Figure 5). Subsequently, the protein level of COL2, ACAN, MMP13, and MMP13 was tested by the WB experiment, and the results of the WB experiment suggested similar results that the expression of the ECM‐related protein was decreased after being induced by H_2_O_2_ (Figure 6). Our research demonstrated that Fis could partly inhibit H_2_O_2_‐induced ECM remodeling in NPMSCs.

### 3.6 EX‐527 reversed the protective effect of Fis on H_2_O_2_‐induced NPMSCs

**Figure 7:**
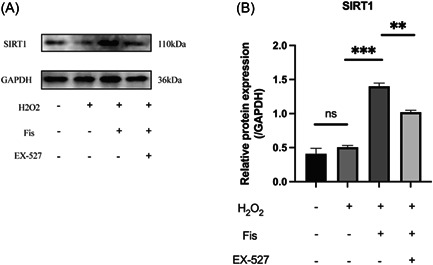
(A) The result of Western blot analysis. (B) The protein expression of SIRT1 has been activated in NPMSCs after stimulated with Fis, and this protective effect can be partly inhibited by EX‐527. *p < .05, **p < .01, ***p < .001, ****p < .0001. NPMSC, nucleus pulposus mesenchymal stem cell; ns, no significance.

The potential mechanism of the protective impact of Fis on biological effects caused by H_2_O_2_ in NPMSCs has been studied in this study. We assumed that the Fis is an activator of SIRT1 and hypothesized that the protective effects of Fis in NPMSCs were related to SIRT1. Thus, we divided our study into four groups, and the fourth group was NPMSCs cultured with H_2_O_2_, Fis, and EX‐527 (a specific inhibitor of SIRT1). We tested the SIRT1 protein expression using WB experiment, the results showed that the SIRT1 might be involved in the protective effects of Fis (Figure 7). And the protective effects of Fis were partly inhibited, indicating that the protective effects of Fis on NPMSCs may relate to SIRT1.

## 4 DISCUSSION

It has been accepted that LBP is a major skeletal muscle disorder, which poses a high personal, social, and economic burden.^13^ Although a series of etiologies may lead to LBP, including trauma, infection, and tumor.^14^, ^15^, ^16^ IDD has been regarded as the most crucial reason for LBP. It has been reported that more than 40% of LBP is associated with IDD.^17^ Until now, the mechanism of IDD is not fully clear, so we must illuminate the pathology of IDD and find new effective reagents for IDD treatment.

The IVD connects the adjacent vertebral, which plays a vital role in maintaining the stability and flexibility of the whole spine. The physiology dysfunction of IVD caused by multiple stress may eventually lead to LBP.^18^ Currently, the molecular basis and pathogenesis of IDD have not been completely understood, and surgical and conservative treatments can only relieve the clinical symptoms but not inhibit or reverse the process of IDD.^19^, ^20^ Therefore, investigating the mechanism of IDD is necessary.

As one of IDD's most promising and appealing regenerative strategies, stem cell‐based therapy has achieved great advances in treating different areas of degenerated diseases and IDD.^21^, ^22^ In IDD research, other kinds of stem cells also have been studied, including BMSCs, and ADMSCs.^23^, ^24^ The results of this research were promising. In recent years, more research has focused on endogenous multipotent cells in IVD. The NPMSCs exist in the inner IVD and with the ability to differentiate into chondrogenic cells, adipogenic cells, and orthogenic cells after specific stimulation.^25^, ^26^, ^27^ There is a strong differentiation ability of NPMSCs compared with BMSCs and ADMSCs for NP‐like cells. Thus, the research of NPMSCs in treating IDD has gotten more attention for its potential to supply the NP cells decreased in the IVD, and it has been aimed as a new and effective idea for IDD treatment.^8^

The harsh microenvironment and metabolic imbalance in the inner IVD have been identified as the main cause of the reduction of the number and function of NPMSCs.^25^ Thus, it is vital to explore the potential therapy of inhibiting the harmful effects of oxidative stress on NPMSCs. As for this aim, we designed and conducted this study.

Sirtuins, a family of nicotinamide adenine dinucleotide (NAD^+^)‐dependent histone deacetylases, are critical regulators in aging‐related diseases.^28^, ^29^ SIRT1 is known as a target regulator in protecting against oxidative stress, inflammatory response, and cell apoptosis.^30^, ^31^, ^32^ According to IDD research, SIRT1 has been reported to be involved in cellular homeostasis and is a promising agent in IDD treatment.^33^, ^34^ It has been reported that during the progression of IDD, the SIRT1 mRNA level decreases.^35^ Guo et al.^36^ revealed that the Pfirrmann grade of IVD is negatively correlated with the SIRT1 protein expression. In addition, Song et al.^37^ showed that DHP (an activator of SIRT1) could activate the SIRT1 in the NP cells and inhibit IL‐1β‐induced oxidative stress. Thus, focusing on the SIRT1 activator may be a novel and promising therapy for IDD.

Fis has been tested as an activator of SIRT1, and the effects on SIRT1 have been found in many studies.^38^, ^39^ A recent study found that Fis activates SIRT1 to ameliorate oxidative glutamate testicular toxicity.^40^ Also, it has been reported that Fis ameliorates alcohol‐induced liver injury by regulating SIRT1.^41^ However, the potential protective effects of Fis on NPMSCs are unclear, and the mechanism of Fis function in NPMSCs is significant for us to research.

This study detected firstly that Fis protected NPMSCs from apoptosis, inflammation, and oxidative stress caused by H_2_O_2_ and the possible molecular mechanisms. NPMSCs from rat NP tissue were successfully isolated and cultured. It was revealed that 10 μM of Fis was safe for NPMSCs and might minimize cell viability reduction caused by H_2_O_2_. It was shown that the protective effects of Fis on NPMSCs were found in cell apoptosis, inflammatory response, and ECM degradation. EX‐527 could partly inhibit all these protective effects, and it was concluded that Fis's potential mechanism might be related to SIRT1. However, there were some limitations of our study, and one was that we only tested the hypothesis in a cell model, and the animal model had not been established. In addition, the microenvironment in the IVD is hypoxic, perhaps culturing cells derived from intervertebral disc under hypoxic conditions can better simulate the environment inside IVD theoretically. And compared to GAPDH, HPRT is an enzyme that may play a role as a superior housekeeping gene to use in tissue culture that involves alterations in oxygen levels and ROS.

## 5 CONCLUSION

In conclusion, this study demonstrated that Fis attenuated apoptosis and inflammation of NPMSCs induced by oxidative stress in‐vitro. The present results also showed that inhibition of SIRT1 may decrease the effects of Fis on NPMSCs, indicating SIRT1 may involve in this pathology. The results added the theoretical basis for research on new treatment of IDD based on NPMSCs.

## AUTHOR CONTRIBUTIONS

**Qing Zhou**: Conceptualization; data curation; funding acquisition; investigation; project administration; visualization; writing—original draft; writing—review and editing. **Chao Zhu**: Investigation. **Anwu Xuan**: Investigation. **Junyou Zhang**: Investigation. **Zhenbiao Zhu**: Investigation; visualization. **Liang Tang**: Investigation; visualization. **Dike Ruan**: Funding acquisition; project administration; supervision; writing—review and editing.

## CONFLICT OF INTEREST STATEMENT

The authors declare no conflict of interest.

## ETHICS STATEMENT

Animal experiments were approved by Institutional Animal Care and Use Committee (approval number AWE2021122401). The experimental process strictly followed the approved protocol.

## Data Availability

Data sets will be available from the corresponding author upon reasonable request.
